# Recent Advances in Mycotoxin Determination in Fish Feed Ingredients

**DOI:** 10.3390/molecules28062519

**Published:** 2023-03-09

**Authors:** Sofia Vardali, Christina Papadouli, George Rigos, Ioannis Nengas, Panagiota Panagiotaki, Eleni Golomazou

**Affiliations:** 1 Department of Ichthyology and Aquatic Environment—Aquaculture Laboratory, School of Agricultural Sciences, University of Thessaly, 38446 Volos, Greece; 2 Institute of Marine Biology, Biotechnology and Aquaculture, Hellenic Centre for Marine Research, 46.7 km Athens-Sounion, 19013 Attiki, Greece

**Keywords:** mycotoxins, aquafeeds, aquaculture, cereals, detection, HPLC, ELISA, FT-NIR, biosensors

## Abstract

Low-cost plant-based sources used in aquaculture diets are prone to the occurrence of animal feed contaminants, which may in certain conditions affect the quality and safety of aquafeeds. Mycotoxins, a toxic group of small organic molecules produced by fungi, comprise a frequently occurring plant-based feed contaminant in aquafeeds. Mycotoxin contamination can potentially cause significant mortality, reduced productivity, and higher disease susceptibility; thus, its timely detection is crucial to the aquaculture industry. The present review summarizes the methodological advances, developed mainly during the past decade, related to mycotoxin detection in aquafeed ingredients, namely analytical, chromatographic, and immunological methodologies, as well as the use of biosensors and spectroscopic methods which are becoming more prevalent. Rapid and accurate mycotoxin detection is and will continue to be crucial to the food industry, animal production, and the environment, resulting in further improvements and developments in mycotoxin detection techniques.

## 1. Introduction

The aquaculture industry has become a source of high-quality protein providers for humans worldwide [1]. It is one of the fastest-growing industries in food production with an average growth rate of 5.3% between 2001 and 2018 [1], globally accounting for more fish biomass than capture fisheries [2]. Fish stock depletion, rapid increase in global population, high demand for seafood products, and international trade have contributed to the tremendous aquaculture expansion during the past decades [3]. The challenge of the aquaculture industry to meet increasing fish demand and achieve food security goals within environmental boundaries will become critical in the coming years, especially with a global population headed to 10 billion by 2050 [4,5].

Marine ingredients are highly important in aquatic feed to provide macro- and micro-nutrients and organoleptic properties, enhancing the digestibility and growth performance of formulated diets. Marine ingredients used in aquafeed are usually meals and oils rendered by small pelagic fish, and by-products of fish and seafood processing [6]. Fish meal is considered the most valuable protein source, due to its exceptional benefits including its well-balanced composition of amino acids, good digestibility, and palatability, as well as its enhancement of the digestion, and absorption of nutrients in fish diets [7].

About 70% of aquaculture production depends on providing aquatic animals with high-quality, rich in protein aquafeeds [1]. Aquaculture globally is currently consuming around 69% of fishmeal and 75% of fish oil supplies [8] and as it is continuously expanding, the demand for fishmeal and fish oil produced by marine pelagic fisheries will be steadily increasing. This has led to a progressive decline in these fish stocks and caused severe inflation in their global prices [9]. Given the increased demand for high-quality fish species, aquaculture addresses the challenge of succeeding in sustainable growth by replacing fish protein sources with plant and terrestrial animal proteins, without compromising the economic value and quality of the final product [10].

Plant-based protein sources have been generally used to partially substitute fish meal in fish diets [11]. These protein sources are advantageous by having a high content of available protein, continuous availability, environmental sustainability, and affordable prices [12,13,14]. Plant-based feed ingredients also provide dietary carbohydrates which can be a source of energy for fish and shrimp; depending on their ability to utilize dietary carbohydrates for energy depending on the species and their natural diet [15]. Furthermore, plant ingredients are sources of starch which is necessary as a binder and facilitates extruded pellet expansion [16].

Plant-based feed ingredients currently used in aquafeeds as substitutes for marine ingredients include among others, soybean meal, rapeseed/canola meal, maize/corn, wheat bran, wheat, and barley [17]. Corn gluten is also a promising ingredient in fish feed, due to its high nutrient content and its increased availability as a bioethanol production by-product [18]. Corn gluten and wheat gluten are high in protein, low in fiber, rich in vitamins B and E, and do not contain any antinutritional factors [19]. Sunflower meal is highly palatable and has low antinutritional factors [20]. Soybean meal is one of the most interesting alternatives to fishmeal because of the advantages of easy supply, low price, and increased protein and amino acid composition [21]. However, soybean meal has been found to induce a variety of histological and functional changes in the gastrointestinal tracts of several species, such as subacute enteritis of the distal epithelial mucosa including morphological alteration and inflammation [22].

As opposed to the aforementioned benefits of plant-based sources, animal diets based on plant proteins can be often associated with reduced feed intake, growth performance, and intestinal function [13,22,23]. Some ingredients of plant origin have certain characteristics, such as high carbohydrate content, deficiency in some essential amino acids, low palatability, as well as content in some anti-nutritional factors [24], that limit their use. Both the quality and safety of aquaculture diets can be affected by anti-nutritional compounds including phytates, protease inhibitors, saponins, glycosylates, and tannins, resulting from the inclusion of plant dietary sources [25]. Fish diets may be also contaminated by mycotoxins, also derived from plant-based raw materials [26].

Mycotoxins are secondary metabolites produced by various species of fungi, often found in agricultural products that are used to feed livestock. These toxins pose a health risk to both livestock and consumers. Agricultural raw materials can be contaminated by fungi during the growing process, before harvest, or during storage in inadequate conditions of humidity and/or temperature [25]. Since mycotoxins are natural contaminants and pose a health risk to both livestock and consumers, several European and international organizations have dealt with this issue by identifying their particular importance and establishing regulatory limits and proposing recommended levels for selected mycotoxins. These include the European Commission (EC, Brussels, Belgium), the US Food and Drug Administration (FDA, Silver Spring, MD, USA), the Food and Agriculture Organization of the United Nations (FAO, Rome, Italy), and the World Health Organization (WHO, Geneva, Switzerland). A scientific expert committee jointly convened by WHO and FAO, named JECFA, serves actually as the international body responsible for evaluating the health risk from all natural toxins including mycotoxins.

Several mycotoxins have been identified and those of significant importance in animal feeds are primarily produced by the five fungal genera *Aspergillus*, *Fusarium*, *Penicillium*, *Claviceps,* and *Alternaria* [27]. Approximately 400 compounds have been identified as mycotoxins [28]. Mycοtoxins may cause health issues in livestock when accidentally present alone or synergistically in animal diets [29]. The aflatoxins (AFs) such as aflatoxin B1, B2, G1, G2, and M1 are human and animal health hazards according to the International Agency for Research on Cancer (IARC) [30]. Moreover, ochratoxins (OTA), and fumonisins (FBs) B1, B2, and B3 have been assessed as possible human carcinogens [31,32]. Furthermore, other mycotoxins have been considered as serious threats including trichothecenes (TCs) type A (HT-2 toxin and T-2 toxin) and B (deoxynivalenol-DON), zearalenone (ZEN), Fusarium mycotoxins, ergot alkaloids (EAs), Alternaria toxins (ATs) and patulin (PAT) [33].

AFB1, DON, ZEN, and FB1 belong to the most contaminants of animal feeds [34]. Mycotoxic contamination may considerably affect animal health, causing functional abnormalities, toxicity hepatic problems, immunotoxicity issues, and reduced growth and animal productivity [35,36,37,38,39]. ZEN may induce reproductive problems such as hyperestrogenism, sterility, and even abortions, affected by the estrogenic activity of ZEN which interferes with animal reproduction [40]. In fish feed, FBs and DON are among the most frequently detected mycotoxins at high levels [26]. These mycotoxins can potentially cause problems in fish farm operations, with significant economic losses such as mortality, reduced productivity, and higher susceptibility to diseases [39].

AFB1 is the only mycotoxin regulated by Directive 2002/32/EC of the European Parliament and of the Council of 7 May 2002 on undesirable substances. The maximum allowed concentration in feed materials for fish species is 20 µg/kg (ppb), and for complete feed is 10 ppb [41]. Concerning other important mycotoxins such as DON, ZEN, T-2 and HT-2 toxin, FB1 and FB2, the European Commission (EC) has established only recommended limits for their presence in feedstuffs and feed [42,43,44]. Among these recommended limits, only values for FB1 and FB2 refer directly to fish species. The recommended maximum concentration for DON is 8000 ppb for cereal and cereal products except for maize by-products, while for complementary and complete feeding stuffs the limit is 5000 ppb. For ZEN, the recommended limits are 2000 ppb for cereals and cereal products except for maize by-products. For OTA, the limit is 250 ppb for both cereals and cereals products. For the summary of FB1 and FB2, EC has proposed the limit of 10,000 ppb for complementary and complete feeding stuffs for fish. For T-2 and HT-2 toxins in cereals and cereal products, except for oat bran, the recommended limit is 500 ppb, while for ergot alkaloids found in feed containing unground cereals, the limit is 1000 ppb.

The potential hazards of mycotoxin presence in feed materials have driven efforts to develop various analytical methods for the identification and quantification of mycotoxins in food samples. Continuous improvements in mycotoxin analytical methodology using advanced and rapid techniques are paramount to comply with the updated legislation and protect consumers of aquatic products. Recently, the existed methodologies for myco-toxin detection related to human and animal health were reviewed in different food matrices [45,46]. The present review focuses primarily on the advances in mycotoxin detection during the last decade in plant-derived raw materials comprising the major fish feed ingredients. Extraction and analytical methods are briefly covered as well as consideration for the future of mycotoxin analysis.

## 2. Sampling and Sample Preparation Methods

### 2.1. Sampling and Sample Preparation

In the mycotoxin sampling process, it is essential to ensure accuracy and representativeness in the sample collection. Mycotoxins can be found in marginally detectable amounts, and products are not uniformly contaminated, increasing the risk of inaccurate sampling [47,48]. A specific protocol is followed to collect representative laboratory samples from all sampling points. The lots selected for inspection must be appropriate. The final sample is the combination of several replicate samples from different parts of the lot. This is achieved by mixing and dividing to obtain a representative sample [49,50].

Mycotoxin detection is typically done by collecting and testing samples of food or feed. The samples should be taken randomly from the entire lot and should be collected from different locations within the lot [50]. The sample size needed depends on the size of the lot, but a minimum of 500 g should be collected using clean and properly sterilized sampling equipment to avoid contamination [51]. Samples should be stored in airtight containers, at the appropriate temperature and humidity, and transported to the laboratory as quickly as possible to prevent degradation of the mycotoxin [51]. The goal of sample preparation is to create a representative and clean sample that can be accurately analyzed by the chosen analytical technique. The specific steps involved in sample preparation depend on the type of sample and the analytical technique used. To obtain an analytical part (test portion), a sample must be ground, homogenized, and subsampled. This analytical portion is then extracted with a solvent, analyzed, and the mycotoxin concentration is determined using a validated analytical approach [52].

Sample preparation is an important step in analytical techniques because it could greatly affect the accuracy and precision of the results.

### 2.2. Sampling Error

Sampling errors can occur in several ways and can greatly affect the accuracy and precision of analytical results. Sampling errors in mycotoxin detection refer to the potential for variability in the results of mycotoxin testing due to the collection and preparation of the sample [53]. Such errors may also occur when a sample is not representative of the entire lot, or when the sample is contaminated during collection or handling [54].

To minimize the sampling error, it is important to follow proper sampling procedures, including the selection of multiple samples from different locations within the batch and using appropriate sampling tools and techniques [55]. Additionally, samples should be properly stored and prepared according to established protocols. Quality control measures should be also implemented to ensure the accuracy and reliability of the testing results [56].

### 2.3. Sample Pre-Treatment (Extraction and/or Clean-Up)

#### 2.3.1. Solid–Liquid Extraction (SLE)

Solid–liquid extraction involves a simple and high-sensitivity process for preparing samples in liquid chromatography–mass spectrometry (LC-MS). The multi-residue analysis ability of the detection apparatus is rather advantageous [50,57]. The capacity of matrix effects in LC-MS analysis to modify chromatographic signals is accomplished due to co-eluting matrix components, while the ion suppression problem that arises can be overcome by proper sample preparation using specific matrices and internal standards [57]. Apart from being simple and sensitive, sample preparation in this procedure is considered reliable and has been successfully applied to detect mycotoxins in plant samples. Few methods were found in pertinent literature using simple SLE extraction without any further clean-up steps. All methods used a solvent mixture of acetonitrile/water for the extraction of mycotoxins before LC-MS/MS analysis [58,59,60,61]. However, the simplicity of sample preparation process may affect the method’s performance characteristics (low recoveries, matrix effect, etc.,) [62,63,64].

#### 2.3.2. Dispersive-Solid Phase Extraction (d-SPE)

In the d-SPE technique, sample cleaning is achieved by the use of a solid sorbent in a liquid or dissolved sample which retains impurities. After separation, the sample is centrifuged for sorbent removal (Anand and Srivastava, 2020). Different types of sorbents are used. C18 sorbent is used to extract non-polar or relatively polar compounds, retaining most of the organic compounds present in an aqueous phase.

The QuEChERS protocol as named by quick, easy, cheap, effective, rugged, and safe, is a commonly applied extraction method that requires small amounts of sample and solvent while at the same time producing high extraction efficiency. This advantage explains its high popularity in current extraction techniques [55]. The extraction phase is the first step where an organic solvent such as acetonitrile is needed along with a variety of salts to modulate polarity and pH and to facilitate phase separation and recovery of the analyte. Purification is the second stage in the cleaning process. The remaining water and other interfering substances from the matrix are eliminated in this stage [65]. There are several methods using the QuEChERS protocols for the extraction and clean-up of mycotoxins before instrumental analysis [66,67,68,69,70,71,72].

#### 2.3.3. Clean Up by Immunoaffinity Column (IAC)

The creation of IAC is another technique where specific antibodies for certain mycotoxins are bound to a specifically activated SP support. This method is commonly used to detect Afs, OTA, and FBs. In detail, the support is packed into a cartridge while a suspension is performed in a buffer solution. After the extract or fluid’s mycotoxin attaches to the antibody and any contaminants are washed away with water or an aqueous solution, the mycotoxin is desorbed using a miscible solvent (methanol). IAC can be used for further separation and LC quantification [73]. A few methods were found in the literature using the IAC columns for mycotoxins clean-up step [74,75,76,77].

#### 2.3.4. Solid Phase Extraction (SPE)

The SPE reduces matrix-based interferents to concentrate on a target analyte. To properly separate the analytes from the other interferents, this absorbent is selected based on the physicochemical characteristics of the analytes [78]. Dispersive SPE, is a modern technique that requires nanoparticles in a magnetic mode. This detection method has been recently adopted [79]. Notably, the characteristics of the magnetic SPE (mSPE) resemble those found in standard SPE. Matrix composition may affect the selection of the adsorbent and elution mixture [79]. In mSPE, continuous contact with the adsorbent is necessary through the dispersion of the magnetic material into the solution containing the target molecules.

A method using modified magnetic nanoparticles as a solid phase adsorbent for extraction of OTA in rice, wheat, and corn has been developed and very low limits of detection were achieved (0.03–0.06 μg/kg) while the recoveries are 87 to 93% [80]. Furthermore, another method using mSPE as a clean-up step has been developed for the determination of AFB1, AFB2, AFG1, AFG2, OTA, and ZEN in various kinds of cereal using a LC-MS/MS system [81].

#### 2.3.5. Molecular Imprinted Polymer (MIP)

For clean-up and preconcentration of mycotoxins, a new class of intelligent polymers based on MIPs has proven to be an effective technique. The MIP is a synthetic material with an artificially generated three-dimensional network that can specifically rebind a target molecule. MIP is cost-effective, chemically, and thermally stable and compatible with all solvents [82]. A magnetic MIP (mMIP) with quercetin as a dummy template has been used for the extraction of ZEN from maize, wheat, and rice by Cavaliere et al. 2019 [83].

#### 2.3.6. Ultrasonic Solvent Extraction (USE)

The USE method involves mechanical wave propagation that makes up an ultrasound created by cycles of compression and refraction, or waves with high and low pressures combined in frequencies above 20 kHz. Temperature and pressure changes may affect USE operation by the creation of bubbles. Both particle collisions and ultrasonic waves can cause fragmentation, which decreases particle size and aids in mass transfer [84]. The combination of USE with SLE extraction is used in an LC-MS technique for mycotoxin detection [75].

## 3. Instrumental Analysis

### 3.1. Chromatographic Methods—Detection Systems

Chromatographic-based methods include liquid chromatography (LC) or gas chromatography (GC) that is coupled with ultraviolet (UV), mass spectrometry (MS), or fluorescence (FLD). The chromatographic methods combined with a UV detector and FLD are usually used for the analysis of a compound or a small number of mycotoxin-related chemicals. The MS method has many advantages such as high sensitivity, selectivity, and accuracy, compared to the two other methods. Tandem MS (MS/MS), where two MS equipment are coupled together, is a highly sensitive, specific, and reliable tool for detecting contaminants in food and has become the most popular approach for multianalyte analyses [85,86]. LC-tandem MS (LC-MS/MS) has been increasingly used for the accurate quantitative analysis of mycotoxins in food [87]. A limited number of multi-mycotoxin techniques, particularly for finished fish feeds and shrimp feeds, has been reported. Aquatic feeds are complex matrices consisting of minerals, vitamins, fatty acids, and proteins in high concentrations that are challenging to remove [88]. As a result, choosing an appropriate clean-up step that reduces matrix effects (MEs) and interferences during chromatographic analysis is essential [89]. The literature on exclusive analyzes of fish feed ingredients seems insufficient, therefore, the data are mainly based on analyses of raw materials used in all types of animal feed, including fish feed. Table 1 summarizes the methods reviewed mainly in the past decade using HPLC systems equipped with several detection systems for the determination of mycotoxins in fish feed ingredients and aquafeeds.

Most of the methods found in the literature use LC-MS/MS as a detection system. Some of them using various extraction processes before LC-MS/MS analysis for the mycotoxin determination are detailed below. LC-MS/MS method was reported for the analysis of 15 mycotoxins in fish feed. The extraction was achieved by a USE step followed by a clean-up step by a lipid cartridge. The recoveries varied between 25 and 109% for the 15 mycotoxins and LODs ranged between 0.05 and 54 μg/kg [101]. Furthermore, for the detection of AFB1, AFM1, T-2, HT-2, DON, OTA, and ZEN in fish feed and shrimp feed, an HPLC-MS/MS method was developed. The samples were extracted with a mixture of acetonitrile and water followed by a defatted step by hexane and a clean-up step with a multi-toxin purification column. LODs ranged between 1.83 and 12.63 μg/kg and the method was successfully applied in several fish feeds in China [88]. The LODs of the method ranged between 0.15 and 61 μg/kg [59]. Another method for the simultaneous determination of ZEN and DON in fish feed was reported based on an HPLC-DAD system and an SLE extraction step followed by clean-up with an IAC column [100].

Concerning fish feed ingredients, the simultaneous determination of 11 mycotoxins in maize, wheat, and barley was achieved by UHPLC-MS/MS analysis using a simple SLE extraction with acetonitrile/water/formic acid (79/20/1, *v*/*v*/*v*). The UPLC-MS/MS method for the analysis of DON and T-2 in maize and oats was also developed, followed by SLE extraction and an SPE clean-up step. The LODs of the method were between 0.13 and 0.38 μg/kg [93]. In wheat, corn, rice, and barley, LC-MS/MS was applied for the determination of 38 mycotoxins using a QuEChERS extraction [70]. Another LC-MS/MS method was developed for the detection of T-2 and HT-2 toxins in corn and oat using an SLE extraction followed by a clean-up step with an IAC column, achieving very low LODs, ranging between 0.02 and 0.08 μg/kg [74].

Only four methods were found using an FLD detection system. In rice, wheat, and corn samples, the mSPE extraction before LC-FLD detection was applied for the analysis of OTA with LODs ranging from 0.03 to 0.06 μg/kg [80]. For the detection of aflatoxins AFB1, AFB2, AFG1, AFG2, and DON in maize, oat, rice, rye, barley, and wheat an HPLC-DAD-FLD system was developed, after two SLE extractions and a clean-up step by SPE. The LODs of the method were 0.02 to 16.2 μg/kg [95]. An automated molecularly imprinted SPE system with online fluorescence detection MISPE-FLD was applied for the determination of OTA in wheat samples achieving LOD 1.2 ng/mL [96]. In wheat, corn, oat, barley, and rice, a validated HPLC-FLD system coupled with a photochemical reactor was tested for the simultaneous determination of aflatoxins, OTA, and ZEN. The LODs of the method ranged between 0.004 and 0.5 μg/kg [99]. Full scan MS was also found in pertinent literature using a TOF-MS and an Orbitrap MS system for the identification of mycotoxins [61,71,94]. Orbitrap MS and TOF MS are used to estimate both known and unknown compounds. This is because they have the ability to allow detailed discrimination in molecular weight by accurately measuring the mass to five significant digits [71]. A validated UHPLC-ToF-MS method was developed for the determination of nine mycotoxins in maize. The extraction step proposed was very easy, using two SLE steps with acetonitrile 80% (*v*/*v*). The method’s LODs ranged between 0.5 and 62.5 μg/kg [61]. Furthermore, the LC-Orbitrap MS method combined with QuEChERS step was applied for the determination of 20 fusarium toxins in corn, wheat, barley, sunflower, soybean, feeds, and feedstuffs and the LODs of the method were 5 μg/kg [68]. Ten mycotoxins in maize and rice were detected by a full-scan LC-MS method using a second-order calibration method based on an alternating trilinear decomposition (ATLD) algorithm. The extraction was achieved using a USE extraction after the addition of MeOH/H_2_O/CHCl_3_ (75:20:5, *v*/*v*/*v*) and NaCl. The LODs of the method ranged between 0.01 and 1.17 μg/kg [94].

A UPLC method coupled with a photo diode array detector (DAD) for the analysis of T-2 and HT-2 toxins in oats and wheat has been also evaluated. The extraction solvents used were methanol/water (90:10, *v*/*v*) followed by a clean-up step with an IAC column. The LOD of the method for the two toxins was 8 μg/kg [76].

### 3.2. Immunological Methods (Enzyme-Linked Immunosorbent Assay-ELISA)

Lateral flow immunoassay, ELISA, and immunosensors are immunochemical detection methods based principally on antibody–antigen binding [102]. Antibodies and antigens belong to some of the most commonly used capture agents in immunoassays for disease treatment, environmental monitoring, and food safety regulation. Their high commercial recognition is, however, not deprived of drawbacks. For example, immunization and purification are necessary for the development of high-quality antibodies. These processes can be difficult, expensive, and laborious. Additionally, the applicability of antibodies is limited due to their sensitivity to pH and temperature variation. Moreover, antibodies can only recognize substances that are immunogenic and immunoreactive. Finally, the chemical conjugation effectiveness of mycotoxins to a protein carrier is limited [103,104].

Immunosorbent assays and immunosensors require simpler sample pre-treatment compared to those required for chromatographic methods and have the advantages of high throughput and good specificity although, detection results still must be output by instruments. Under the same sample pre-treatment procedure, ELISA assays are however more prone to more errors due to the tedious operation process [105]. Therefore, developing a more sensitive and rapid on-site detection assay is urgently needed to detect toxic and harmful substances in food. Several studies using a variety of assays and detectors have been reported in the literature and are presented in Table 2.

A silver nanoparticle/carbon dot has been applied to develop a “turn on’’ pattern fluorescence quenching FLFIA (fluorescence lateral flow immunochromatographic assays) method for the qualitative and semi-quantitative detection of ZEN in maize, and wheat. This assay had a limit of detection (LOD) of 1–2.5 μg/kg for ZEN in cereal samples [105]. For ZEN detection, a monoclonal antibody (mAb) (2B10) was also prepared. The specific mAb showed no cross-reactivity with other groups of mycotoxins. A competitive microarray assay based on a novel solid supporting material, an integrated poly dimethylsiloxane (iPDMS), was proposed for qualitative and/or semiquantitative determination of ZEN providing a very low limit of quantification (LOQ) 1.02 μg/kg in cereal samples [120]. The variable domain of heavy-chain antibodies (VHHs) as alternative compounds to produce anti-idiotypic antibodies, which work as non-toxic surrogate reagents in immunoassay has also been applied. The proposed method proved to be reliable for the determination of ZEN in cereal samples with a LOD of 6.5 pg/mL. The use of antiidiotypic VHH phage as a non-toxic surrogate and the signal-amplification function of PCR makes it a promising method for actual ZEN analysis in corn, wheat, and rice [117]. In the same ingredients, three kinds of lateral flow immunochromatographic assays (ICAs) using colloidal gold, quantum dots, and polystyrene microspheres have also been used as labels for the detection of ZEN. The assays allow ZEN to be quantified within 20 min with LODs ranging between 6 and 60 μg/kg [123].

For sensitive detection of FB1 in maize, an immunoassay using single-molecule fluorescence correlation spectroscopy was developed. In comparison to conventional ELISA, this method showed high sensitivity, simplicity, a short analysis time, and low reagent and sample requirement. The LOD of this method for FB1 was 1 mg/L [113].

A single-step assay has been developed for the rapid detection of AFB1 in maize, rice, and hazelnut within 15 min with a LOD of 70 pg/mL. For this method, anti-immunocomplex (anti-IC) antibodies were used and the immunoassay was non-competitive showing the applicability of these parameters in the analysis of small molecule contaminants [110]. Furthermore, a chromatography-free method was found in the literature for the detection of AFB1 in cereals and oils through atomic absorption spectroscopy (AAS) using quantum dots and immunomagnetic beads. A magneto-controlled pre-treatment platform for automatic purification, labeling, and digestion was constructed and AFB1 detection through AAS was enabled using the proposed immunoassay which exhibits high sensitivity for AFB1 detection in wheat and maize, with a LOD of 0.04 µg/kg [119].

For the quantitative and simultaneous detection of different mycotoxins, various immunological methods have been assessed. Detection of ZEN, FB1, DON, and AFB1 in corn, and wheat, has been achieved by a suspension array. Suspension arrays have the advantages of sensitivity, rapidity, and accuracy. Signal responses are observed using red and green laser lights to achieve qualitative and quantitative detections. The LODs of the method were 0.51–6.0 ng/mL for the four mycotoxins [111]. Multiplex fluorescent immunosorbent assay (FLISA) using quantum dots (QDs)-based immunochemical techniques has been used for multi-contaminated cereal samples, allowing the simultaneous determination of all compounds. The mycotoxins DON, ZEN, AFB1, T-2, and FB1 were allocated to different wells of the same multi-well plate, and the sample was treated before being dispensed over the wells (single-analyte multiplex, SAM). Moreover, multi-contamination with ZEN and AFB1 was determined with the double-analyte multiplex (DAM). Two different specific antibodies were distributed in one single well and the mycotoxins ZEN and AFB1 were determined in wheat and maize, on the condition that their conjugates are labeled with QDs, which are fluorescent in different parts of the spectrum at two different wavelengths [121].

Another sensitive tool for the simultaneous quantitative determination of DON, ZEN, and AFB1 in cereal-based products in one single well of a microtiter plate, has been applied. This one is based on the use of a colloidal quantum dot enrobed into a silica shell (QD@SiO2) derivatives as a highly responsive label. Silica-coated quantum dots were prepared and subsequently modified via co-hydrolysis with tetraethylorthosilicate (TEOS) and various organosilane reagents. The LODs were 1.9–5.4 μg/kg for the three mycotoxins [114]. Moreover, another study proposed the development of a polyvinylidene fluoride (PVDF) membrane-based dot immunoassay for the rapid and simultaneous detection of AFB1, ZEN, DON, OTA, and FB1 in corn, and wheat. The LODs of the method for mycotoxins were 20–1000 μg/kg [116].

Cereal contamination with ZEN and DON, was identified using Cd-based QDs as labels, while an imprinted BSA was immobilized on a microwell plate. This technique involved putting silica on green- and red-emitting QDs to turn them hydrophilic, before coupling with mycotoxin-protein occurs. The ZEN detection cut-off level varies depending on cereal origin. On the other hand, the cut-off level for DON is considerably lower when compared to its permissible limits [118]. The multi-mycotoxins (AFB1, ZEN, and T-2 toxin) determination in cereals has also been accomplished using a multicolor immunochromatographic strip (ICS). On this method, three monoclonal antibodies are bound to three different colored nanoparticles to act as immunoassay probes and the three mycotoxins may be quantified at the same time according to color decrease [122].

### 3.3. Biosensors

Biosensors consist of various elements such as a molecularly imprinted polymer (MIP), an aptamer, a DNA/RNA molecule, an enzyme, a tissue, living cells, and antibodies. A transducer is also necessary to connect these parts, which transforms the observed physical or chemical changes into a quantifiable signal. Depending on the signal transduction mechanism and the applied recognition elements, three categories of biosensors exist: optical, electrochemical, and piezoelectric. Immunosensors are of the most commonly used analytical methods for mycotoxin detection, although other cutting-edge methods such as MIP-based sensors are available. Antibodies, antigens, and their fragments, are used for biomolecular recognition in immunosensors. The essential premise behind all immunosensors is that the precise binding of the immobilized components in the sample results in the production of an analytical signal that is affected by the concentration of the target analyte. Labeled and label-free immunosensors combined with different transducers have been considerably developed for mycotoxin assessment [124,125,126]. The various mycotoxin detection sensors that have been created over the previous ten years are presented in Table 3.

For ZEN detection in maize and rice, a fluorometric assay based on mesoporous silica nanoparticles (MSNs-NH2) as a positive charge reactor and an aptamer-FAM (6-carboxy-fluorescein-labeled aptamer) as a signal probe (capture probe and negative charge reactor), was tested respectively. The proposed assay had high recognition specificity, low LOD (0.012 ng/mL), and a wide linear range (0.005–150 ng/mL) [127]. Furthermore, a multi-commutated flow-through optosensor in different cereal samples was developed to quantify ZEN. The mycotoxin was retained and pre-concentrated on C18 silica gel, and the use of the multi-commutated flow manifold allowed the automated retention/desorption of ZEN on the solid microbeads using appropriate carrier/eluting solutions. The native fluorescence of ZEN was recorded on the solid phase at λexc/λem of 265/465 nm/nm. A QuEChERS procedure was used as a clean-up step of ZEN from different cereal samples. Recovery studies were performed to assess the accuracy of the method, obtaining recovery yields between 93% and 107% in all the analyzed samples (maize and cereals feedstuff) and the LOD was 15 μg/kg [128]. Furthermore, a surface-enhanced Raman scattering (SERS)-based test strip was proposed for the detection of ZEN, showing simplicity, rapidity, and high sensitivity. Core-shell Au@AgNPs with embedded reporter molecules (4-MBA) was synthesized as SERS nanoprobe, which exhibited excellent SERS signals and high stability. The detection range of ZEN for corn samples was 10–1000 μg/kg while the LOD of the method was 3.6 μg/kg [130].

DON detection in crop samples (wheat and maize) has been achieved using a white light reflectance spectroscopy (WLRS) optical immunosensor. It was proved to be a fast and high-sensitivity assay for the assessment of contamination in the whole grain [129].

For the determination of the mycotoxin OTA, a differential pulse voltammetric aptasensor based on hybridization chain reaction (HCR) was developed. The assay was successfully applied to the determination of OTA in cereal samples with a detection limit of 2 pg/mL [131]. A portable and reusable optofluidic immunosensor OIP-v2 was developed for rapid and sensitive on-site detection of DON using DON-BSA modified bio-probes as biorecognition elements. The OIP-v2 was used for the detection of DON with high sensitivity, accuracy, and rapidity. The LOD of DON was 0.16 µg/L [132].

### 3.4. Spectroscopic Methods FT-NIR

Infrared (IR) spectroscopy-based methods are the most promising for the detection of mycotoxins since they require small samples and limited technical expertise. Moreover, such techniques are cheap and need no sample pre-treatment. Identification of mycotoxin contamination in crops is commonly carried out using spectroscopic techniques [133]. Mid-infrared (MIR) spectroscopy is specified for molecular vibrations while standard NIR spectroscopy determines the molecular overtones and combined vibrations of chemical bonds. All spectra produced by overtones and mixed vibrations seen in the NIR range are challenging to decipher for specific constituents present in a sample [134]. Chemometrics can be used for direct information extraction from the data, which solves the upcoming necessity of mathematical processing to extract chemicals and linked information in the assessment of NIR and MIR spectra. Three phases are often involved in NIR or MIR spectroscopy using chemometrics: spectral pre-processing, multivariate model construction for calibration, and model transfer [135]. Qualitative and quantitative methodologies are used for NIR and MIR spectroscopic model development. Some examples of qualitative techniques include principal component analysis (PCA), cluster analysis (CA), and linear discriminant analysis (LDA). On the other hand, principal component regression (PCR), multiple linear regression, and partial least squares (PLS) are common methodologies for quantitative multivariate calibration (MLR).

FB1 and FB2 concentrations in maize meal were first analyzed for mycotoxins using FT-IR as a quick way to distinguish contaminated meals [136]. Based on an optimized feature model for NIR spectroscopy, a quantitative assay for AFB1 in maize has been suggested. The potential of NIR spectroscopy in conjunction with chemometric techniques for the quick and accurate quantitative detection of the AFB1 in maize was demonstrated using a portable NIR spectroscopy device to evaluate maize samples with varied degrees of contamination. To effectively mine the wavelengths of the NIR spectra, different variable selection algorithms were used. After the screening, the wavelength variables were utilized to create a support vector machine (SVM) and a partial least squares (PLS) test model, respectively, to measure AFB1 in maize. As a result, by using a nonlinear SVM detection model, the characteristics of NIR spectra are beneficial for the rapid and accurate testing of the AFB1 in maize [136].

NIR spectroscopy is used for rapid ZEN identification in wheat grains. First, using Savitzky–Golay smoothing (SG-smoothing) and multiple scattering correction (MSC), the collected original NIR spectra were denoised, smoothed, and scatter-corrected, before normalized. Random frog (RF), successive projections algorithm (SPA), least absolute shrinkage, and selection operator were the three algorithms utilized to choose variables from the pre-processed NIR spectra (LASSO). In order to achieve the quantitative detection of the ZEN in wheat grains, SVM models were built based on the feature variables extracted by the aforementioned techniques and the LASSO-SVM model’s prediction effect proved to be more accurate [137].

Total FBs (FB1 + FB2) and ZEN in Brazilian maize have also been measured using NIR [138]. There were three regression models used: one for FB1 with 18 principal components (PCs), one for FB2 with 10 PCs, and one for ZEN with 7 PCs. As internal validation, a partial least squares regression technique with full cross-validation was used. When FBs and ZEN were assessed using various assessing calibrations NIR values did not differ significantly compared to reference values LC-MS/MS values, presenting NIR as a reliable method for quick detection of FBs and ZEN in corn [138].

Finally, NIR and FT-NIR were used to evaluate their applicability and efficiency for the analysis of Brazilian wheat flour samples contaminated with DON, with partial least-squares discriminant analysis (PLS-DA) and principal component analysis-linear discriminant analysis (PC-LDA) used as discriminatory methods. Validation samples through PLS-DA showed correct classification rates in the range of 85–87.5% with an error of 10–15% error. For PC-LDA, the hit rate was over 85% with an error of 10–15% demonstrating that NIR is an excellent alternative method for the classification of wheat flour samples according to DON content [139].

## 4. Conclusions and Outlook

The presence of mycotoxins in agricultural products of animal feeds animals poses a health risk to both livestock and consumers. Due to their toxic nature, their prompt detection of mycotoxins is critical to the food industry including aquaculture. During the last decade, analytical methods used for mycotoxin detection include the use of chromatographic, immunological, and spectroscopic (NIR) methods as well as biosensors. Chromatographic methods using LC-MS/MS are more sensitive but require technical competence and higher time investment. Immunological methods such as ELISA and biosensors are less sensitive and reliable but are simpler to use by non-specialized personnel directly in the field and without the requirement of laboratory infrastructure. The FT-NIR spectroscopic method, which has been extensively utilized in recent years for mycotoxin detection, is relatively simple and eco-friendly; however, it requires expensive infrastructure and complex chemometrics and mathematical calculations for its development. As mycotoxins will probably continue to be a problem for the aquaculture industry, to ensure the safety of aquafeeds and produced healthy seafood, it becomes necessary to develop certain detection methods, especially those that can be used on-site.

## Figures and Tables

**Table 1:** HPLC methods used in the analysis of mycotoxins.

Type of Cereal	Mycotoxins	Extraction Process—Clean-up	Analytical Technique	Recovery %	Limit of Detection (LOD)	Ref.
Maize, Wheat, Barley	11 mycotoxins:	SLE acetonitrile/water/formic acid (79/20/1, *v*/*v*/*v*)	UHPLC-MS/MS	63.2–111.2%	0.15–61 μg/kg	[59]
Barley, Wheat, Oat	10 mycotoxins	SLE: 84% (*v*/*v*) aqueous acetonitrile with 1% (*v*/*v*) formic acid Clean-up: d-SPE (mixture octadecyl silica and primary-secondary amine)	UPLC-MS/MS	83.3–92.8%	0.13–3.56 μg/kg	[90]
Barley, Wheat, Oat	23 mycotoxins	QuEChERS Extraction: Acetonitrile 5% formic acid Clean-up: QuEChERS (MgSO_4_ and NaCl)	LC-MS/MS	70.1–109.3%	0.03–2.17 µg/kg	[69]
Corn, Oat	T-2 and HT-2	Extraction: ethanol-water (80:20; *v*/*v*)- Clean-up: IAC	UPLC-MS/MS	78.6–98.6 %	0.02–0.08 μg/kg	[74]
Corn	ZEN, α-zearalenol (α-ZEL), β-zearalenol (β-ZEL), α-zearalanol (α-ZAL), β-zearalanol (β-ZAL), zearalanone (ZAN)	Extraction: acetonitrile/water (90/10; *v*/*v*). Clean-up: SPE using a MycoSep 226 column	Isotope dilution-liquid chromatography/tandem mass spectrometry (ID-UPLC-MS/MS)	96.7−103.6%.	0.14–0.33 µg/kg	[91]
Corn, wheat	T-2, HT-2, diacetoxyscirpenol (DAS) and neosolaniol (NEO)	Extraction: acetonitrile/water, 84/16; (*v*/*v*) Clean-up: SPE withMycoSep 227 column	ID-UPLC-MS/MS	97–103%	0.01–0.12 μg/kg	[92]
Maize, Oat	DON and T-2	Extraction: acetonitrile/water mixture Clean-up: SPE by MycoSep 227 columns	UPLC-MS/MS	85.0–95.3%	0.13–0.38 µg/kg	[93]
Wheat, Corn Rice, Barley	38 (modified) mycotoxins	QuEChERS Extraction: acetonitrile/water/formic acid (75:20:5, *v*/*v*/*v*) d-SPE: anhydrous MgSO_4_, NaCl, Na_2_H-citrate·1.5H_2_O, Na_3_-citrate·2H_2_O	LC-MS/MS	61−120%	LOQ: 0.05−80.0 μg/kg for wheat, 0.07−120 μg/kg for corn, 0.05−150 μg/kg for rice, and 0.10−150 μg/kg for barley	[70]
Maize, Wheat, Rice	ZEN	Extraction: acetonitrile/water, 80:20 (*v*/*v*) with 0.2% HCOOH Clean-up: mMIPs	UHPLC-MS/MS	>95%	0.044 μg/kg	[83]
Maize	AFB1, AFB2, AFG1, AFG2, OTA, ZEN, T2, FB1, FB2	Extraction: 2 SLE steps with acetonitrile 80% (*v*/*v*)	UHPLC-ToF-MS	77.8–110.4%	0.5–62.5 μg/kg	[61]
Corn meal, Durum, wheat flour	AFB1, AFB2, AFG1, AFG2, OTA, ZEN	Extraction: acetonitrile/water/formic acid 80:19.8:0.2 (*v*/*v*/*v*) Clean-up: mSPE	LS-MS/MS	>60%	0.05–2.2 μg/kg	[81]
Wheat flours, Corn meal and other cereal- derived products	AFB1, AFB2, AFG1, AFG2, T-2, HT, FB1, FB2	QuEChERS Extraction: H_2_O 0.1% formic acid, Clean-up: Acetonitrile d-SPE: MgSO_4_ and NaCl	LC-MS/MS	83.6–102.9%	0.5–100 μg/kg	[66]
Maize, Wheat, Sunflower, Soybean, Barley, Feeds, Feedstuffs	22 mycotoxins	QuEChERS Extraction: 2% acetic acid solution, Clean-up: Acetonitrile d-SPE: MgSO_4_ and NaCl	UHPLC-MS/MS	67–94%	0.064–119.04 μg/kg	[68]
Maize, Wheat	11 mycotoxins	SLE extraction: acetonitrile/water mixture	UPLC-MS/MS	52.8–113.9%.	0.08–30.0 μg/kg	[58]
Maize, Rice	10 mycotoxins	USE extraction after the addition of MeOH/H_2_O/CHCl_3_ (75:20:5, *v*/*v*/*v*) and NaCl	LC-MS second-order calibration method based on alternating trilinear decomposition (ATLD) algorithm	93.8–109%	0.01–1.17 μg/kg	[94]
Wheat	10 mycotoxins	QuEChERS Extraction (acetonitrile–water (84/16)) d-SPE: QuEChERS (PSA and C18)	UHPLC-MS/MS	70–116%	LOQ < 7 μg/kg	[72]
Maize, Oat, Rice, Rye, Barley, Wheat	AFB1, AFB2, AFG1, AFG2, DON	Extraction: 2 extractions with water and a mixture of methanol/water clean-up: SPE	HPLC–DAD–FLD	90–112%	0.02–16.2 μg/kg	[95]
Corn, Wheat, Barley	20 Fusarium toxins	Extraction: 2% acetic acid aqueous solution/acetonitrile (1:1, *v*/*v*) clean-up: QuEChERS	LC-Orbitrap MS	71–106%	LOQ: 5 μg/kg	[71]
Barley, Malt	17 mycotoxins	Extraction: (0.1% HCOOH/cetonitrile (1:1, *v*/*v*) Clean-up: QuEChERS (MgSO_4_ + NaCl)	UPLC-MS/MS	75–124% Except of Nivalenol 50–51%	0.3–24 μg/kg	[67]
Rice, Wheat, Corn	OTA	Extraction: SLE Clean-up: mSPE	LC-FLD	87–93%	0.03–0.06 μg/kg	[80]
Rice, Wheat, Oat, Maize, Barley	11 mycotoxins	SLE extraction acetonitrile: water: acetic acid, 79:20:1	UPLC-MS/MS	83.5–107.3%	0.01–25 μg/kg	[86]
Oats, Wheat	HT-2 and T-2 toxins	Extraction: methanol/water (90:10, *v*/*v*) clean-up: immunoaffinity columns	UPLC-PDA	87–103%	8 μg/kg	[76]
Maize, Wheat, Oats, Cornflakes, Bread	14 mycotoxins	Extraction: acetonitrile/water/acetic acid (79/20/1, *v*/*v*/*v*) followed by a hexane defatting step	LC-MS/MS	70–110%	5–13 μg/kg	[77]
Wheat	OTA	Extraction: methanol/3% aqueous sodium bicarbonate (3/7, *v*/*v*) Clean-up: MIP spe column	Automated SPE system with on-line fluorescence detection MISPE-FLD	84–102%	1.2 ng/mL	[96]
Maize, Oats	DON and T-2	Extraction: acetonitrile/water (84:16; *v*/*v*) Clean-up: SPE column	UPLC-MS/MS	85.0–95.3%	0.04–0.12 µg/kg	[97]
Wheat, Maize	35 mycotoxins	QuEChERS: extraction/partition process) of 5% formic acid in acetonitile (MgSO_4_ and NaCl)	UPLC-MS/MS	60–103%	0.13–23.99 µg/kg	[98]
Wheat, Corn, Oat, Barley, Rice	AFB1, AFB2, AFG1, AFG2, OTA, and ZEN	Extraction: 80% methanol Clean-up: multifunctional immunoaffinity column	HPLC-FLD Using a photochemical reactor enhance derivatization system (PHRED)	77–104%	0.004–0.5 μg/kg	[99]
Barley, Oat, Wheat	16 mycotoxins	Extraction: SLE acetonitrile:water:acetic acid (79:20:1, *v*/*v*/*v*)	LC-MS/MS	84–116%	0.1–4.3 μg/kg	[60]
Fish feed and shrimp feed	AFB1, AFM1, T-2, HT-2, DON, OTA, and ZEN	acetonitrile–water (3 + 1, *v*/*v*) saturated hexane clean-up by multitoxin column	HPLC-MS/MS	80.5 to 116.5%	1.83–12.63 lg/kg	[88]
Fish feed	DON and ZEN	SLE Clean-up: IAC column	HPLC-DAD	79–90%	2–30 μg/kg	[100]
Fish feed	15 mycotoxins	USE extraction Clean-up: Captiva EMR Lipid cartridge	LC-MS/MS	25–109%	0.05–54 μg/kg	[101]

**Table 2:** ELISA methods used in the analysis of mycotoxins.

Type of Cereal	Mycotoxins	Method	Detection Method	LOD	Ref.
Maize, wheat, vegetable oil samples	ZEN	Fluorescence quenchometric lateral flow immunochromatographic assay	UV-absorbance	1–2.5 μg/kg	[105]
Maize	FB1	Direct competitive multi-channel immunoassay	Electrochemical	0.58 μg/L	[106]
Oat, wheat, rye, and maize	OTA, DON, FB1 and FB2	Competitive indirect immunoassay	Chemiluminescence	0.9–159 μg/kg	[107]
Wheat and maize	ZEN, T2 and FB1	Competitive assay format	Colorimetric	N/A	[108]
Wheat, Durum wheat, Barley, Maize, Oats	T-2 and HT-2 toxins	Competitive ELISA	Colorimetric	75 µg/kg	[109]
Maize, Rice, Hazelnut	AFB1	Non-competitive immunoassay	Fluorescence	70 pg/mL	[110]
Corn, Wheat, Feedstuff	ZEN, FB1, DON, AFB1	Suspension array immunoassay	Luminex 200 suspension array analyzer	0.51–6.0 ng/mL	[111]
Wheat and corn flours	DON, FB1 and OTA	Magnetic particle-based enzyme immunoassay	Colorimetric	0.1–5 ng/mL	[112]
Maize	FB1	Competitive immunoassay fluorescence correlation spectroscopy (FCS)	Fluorescence	1.0 mg/L	[113]
Maize and wheat	DON, ZEN, AFB1	QD@SiO_2_-based immunoassay	Colorimetric	1.9–5.4 μg/kg	[114]
Wheat, Barley, Soybean, Rice, Maize, Rapeseed meal, Sunflower meal, Complete feeds	ZEN, DON, AFB1 and OTA	Enzyme-linked immunosorbent assay	Colorimetric	1.4–28 μg/kg	[115]
Wheat, Corn, Peanut, Feedstuff	AFB1, ZEN, DON, OTA, and FB1	Polyvinylidene fluoride (PVDF) membrane-based dot immunoassay	Densitometric analysis	20–1000 μg/kg	[116]
Corn, Wheat, Rice	ZEN	Indirect competitive phage ELISA anti-idiotypic VHH phage particles were applied to PD-IPCR	Colorimetric	6.5 pg/mL	[117]
Wheat and maize	DON and ZEN	Multiplex immunosorbent assay	Fluorescence	ZEN:100 μg/kg DON: 700 μg/kg	[118]
Wheat, Maize, Peanut Oil, Husked Rice	AFB1	Quantum dots and immunomagnetic beads	Atomic absorption spectroscopy (AAS)	0.04 µg/kg	[119]
Corn, wheat	ZEN	Competitive immunoassay integrated poly(dimethylsiloxane) (iPDMS)	Chemiluminescence	0.53 μg/kg	[120]
Wheat, Maize	DON, ZEN, AFB1, T2 and FB1 ZEN and AFB1	Fluorescent immunosorbent assay (FLISA) same plate (single-analyte multiplex, SAM) double-analyte multiplex (DAM)	Fluorescence	a. 0.4–10 μg/kg b. 1–1.8 μg/kg	[121]
Maize and cereal-based animal feeds	AFB1, ZEN, T-2 toxin	Multicolor-based immunochromatographic strip (ICS)	Optical	Visible detection limit: 0.5–30 ng/mL,	[122]
Maize, Wheat Rice	ZEN	Three kinds of lateral flow immunochromatographic assays (ICAs)	Colorimetric	6–60 μg/kg	[123]

**Table 3:** Biosensor methods used in the analysis of mycotoxins.

Type of Cereal	Mycotoxins	Method	Detection Method	LOD	Ref.
Maize, Rice	ZEN	Direct binding surface of MSNs-NH2 and the aptamer-FAM (molecular recognition probe)	Fluorescence	0.012 ng/mL	[127]
Maize and cereals feedstuff	ZEN	Flow-through fluorescence sensor	Fluorescence	15 μg/kg	[128]
Wheat and maize samples	DON	Competitive immunoassay	Optical immunosensor White Light Reflectance Spectroscopy (WLRS)	62.5 µg/kg	[129]
Corn	ZEN	SERS-based test strip bimetallic core–shell Au@AgNPs with embedded reporter molecules (4-MBA) as the SERS nanoprobe	Raman spectrometry	3.6 μg/kg	[130]
Corn	OTA	Differential pulse voltammetric aptasensor based on hybridization chain reaction	Electrochemical	2 pg·/mL	[131]
Corn, Wheat	DON	Indirect competitive immunoassay s	Fluorescence	0.16 µg/L	[132]

## Data Availability

Not applicable.
